# Can obtaining informed consent alter self-reported drinking behaviour? A methodological experiment

**DOI:** 10.1186/s12874-015-0032-z

**Published:** 2015-04-24

**Authors:** Lambert Felix, Patrick Keating, Jim McCambridge

**Affiliations:** Department of Social & Environmental Health Research, Faculty of Public Health & Policy, London School of Hygiene & Tropical Medicine, London, UK; Department of Health Sciences, University of York, York, UK

**Keywords:** Informed consent, Self-report, AUDIT, Alcohol

## Abstract

**Background:**

Informed consent is the foundation of the ethical conduct of health research. Obtaining informed consent may unwittingly interfere with the data collected in research studies, particularly if they concern sensitive behaviours that participants are requested to report on. To address gaps in evidence on such research participation effects, we conducted a methodological experiment evaluating the impact of the informed consent procedure on participants’ reporting behaviour, specifically on their self-report of drinking behaviour as measured by Alcohol Use Disorder Identification Test (AUDIT).

**Methods:**

A two arm double blinded randomised controlled trial was used. University students present in London student unions at the time of recruitment were contacted in two phases (an initial run-in phase followed by the main phase). Those providing positive responses to verbal questions: 1) “are you a student?”; 2) “do you drink alcohol?”; 3) “would you like to take part in a brief health survey, which will take around 5 minutes?” were recruited. Participants received one of the two envelopes by chance, with the sequence generated by an online random sequence generator. One contained the participant information sheet, informed consent form and the AUDIT questionnaire (the intervention group), while the other contained only the AUDIT questionnaire (the comparator group). The primary outcome was the mean AUDIT score, which ranges from 0 to 40. The secondary outcome was the proportion of participants in each group scoring 8 or more on the AUDIT, the threshold score for hazardous and harmful drinking warranting intervention.

**Results:**

A total of 380 participants were successfully recruited, resulting in 190 participants in each group, of which 378 were included in the final analysis. There is no evidence of any statistically significant difference between groups in the primary outcome. A statistically significant difference in the secondary outcome was found in the run-in phase only, and not in the main phase, or overall. Moreover, between-group outcome differences between the two phases suggest an important influence of setting on reporting behaviour.

**Conclusions:**

There is no strong evidence that completion of informed consent itself alters self-reporting behaviour with regards to alcohol, though the effect of settings needs to be further studied.

**Electronic supplementary material:**

The online version of this article (doi:10.1186/s12874-015-0032-z) contains supplementary material, which is available to authorized users.

## Background

Informed consent is the foundation of the ethical conduct of health research [1-4]. Studies of informed consent have, for example, examined how much participants actually read the content and later recall it. We are not aware of any study that has investigated whether the informed consent procedure may interfere with the aims of the research being undertaken. Concerns have existed that in certain circumstances it may do so, for example, with Zelen designs being developed for randomised controlled trials (RCTs) [5-7]. In Zelen designs, participants are randomised first and then informed consent is sought later, often to decide whether or not to accept the assigned intervention. Zelen [6,7] suggested that informed consent can provoke anxiety in certain circumstances that may be better avoided. Informed consent is also considered an essential patient safety process while delivering health care interventions [8,9].

Health risk behaviours such as substance use that are commonly collected by self-reported questionnaires are at risk of either being under- or over-reported [10]. Research suggests that anonymity can improve the validity of such data [11,12]. Alcohol consumption appears particularly vulnerable to under-reporting, as in most societies there is a large shortfall between aggregated survey data and sales data that cannot be entirely accounted for by limitations of survey coverage [13]. RCTs are regarded as the gold standard design for answering research questions in an unbiased manner, including in evaluations of intervention effects. Nevertheless, routine procedures including informed consent undertaken in RCTs and other types of studies may diminish impact on participant’s behaviour, cognitions, or emotions [14]. This could, then, result in either under- or over estimation of the intervention effects [14,15].

Research activities such as being interviewed, completing a questionnaire, or being observed can have an impact on participant’s behaviour, on both self-reported and objectively ascertained outcomes [16-18]. These have been widely considered to be manifestations of the Hawthorne effect, though this term has been applied in many different ways and is not helpful when used without specification of content [17]. Research studies are contexts in which there may be subtle pressures to behave in particular ways [15]. Such “demand characteristics” are well known in psychology [19], and little considered more widely, in part because they have been so little studied [20]. Reporting on one’s own behaviour is itself a behaviour and effects of the informed consent procedure on reporting behaviour can manifest in reporting or information bias, potentially undermining the achievement of the aims of any study in which research data is collected by participant self-report. Such problems may afflict RCTs, as well as other types of studies, introducing bias in ways which are overlooked [21].

Reporting on one’s own alcohol consumption offers an interesting target for study in relation to possible effects of the research process. Estimates of population prevalence based on self-report of drinking in general population surveys have long been known to be very much lower than those suggested by alcohol sales data [22,23]. As well as problems with recall, various types of social desirability considerations have been implicated in under-reporting [24]. A socially desirable response occurs when participants tailor their reported attitude or behaviour to conform to their perceptions of what is appropriate, acceptable, or desired by others [24]. For example, in this study, participants were unaware of the true nature of research but were informed that the purpose was to study the harmful effects of alcohol on students using the Alcohol Use Disorder Identification Test (AUDIT). Moreover, the purpose of the study was disclosed only to those allocated to the intervention group (IG) through the participant information sheet. So, we hypothesised that participants in IG would under-report their alcohol consumption in order to provide a socially desirable response.

Unwitting interference with the data collected in research studies, may be a particular concern in relation to personal behaviours about which there are sensitivities, and which participants are requested to report on. To address the many gaps in evidence on “research participation effects”, we conducted this methodological experiment evaluating the impact of the informed consent procedure on participant behaviour, specifically on their self-report of drinking behaviour as measured by the AUDIT [25]. The overarching aim of this study was to test for any effects of completion of an informed consent procedure on self-reported drinking behaviour.

## Methods

We designed this study as a cross-sectional investigation to remove the possibility that participants may change their drinking behaviour in response to AUDIT completion [26]. Our hypothesis was that completion of a standard informed consent procedure would reduce self-reported drinking behaviour and consequences as measured by the AUDIT in comparison with the absence of a consent procedure.

The AUDIT has 10 items covering 3 different aspects of drinking: alcohol use (first 3 items), dependence (next 3 items), and other consequences of drinking (last 4 items) [27]. Each item is scored from 0 (“never”) to 4 (“daily or almost daily” for most of the items) with a possible maximum score of 40. There were two outcomes of interest; the primary outcome was mean AUDIT score and the secondary outcome was the proportion scoring 8 or higher (AUDIT 8+), the conventional threshold for identification of hazardous or harmful drinking.

### Ethical approval

The study was given ethical approval by the London School of Hygiene & Tropical Medicine Research Ethics Committee (LSHTM ethics ref: 6526).

### Study design

We used a two arm double blind randomised controlled trial design to investigate the hypothesis that obtaining informed consent in the standard way prior to the completion of the AUDIT form would lead to decreased self-reported hazardous and harmful drinking in comparison with a comparator group from whom consent was not be obtained. All participants were blinded to the true study purpose, and the fieldworker (LF) was blind to allocation status.

### Participants and setting

Current university students, irrespective of age, gender, year of study, degree, place or subject of study who were present in the following campuses - School of Oriental and African Studies (SOAS), Goldsmiths College, and University of London Union (ULU) at the time of fieldwork were approached to participate.

### Study procedures

Permission for the study was obtained in advance from each student union. Students were approached by the first author (LF) in informal areas of student union premises such as the union bar, café area and refectories. Efforts were made to avoid contamination, in accordance with a dedicated fieldwork protocol. For example, if there was more than one participant present, then only one was approached. Those providing positive responses to the following questions asked verbally were successfully recruited, “are you a student?”, “do you drink alcohol?”, and “would you like to take part in a brief health survey, which will take around 5 minutes?” as well as a willingness to provide a date of birth. Both date of birth and gender were recorded to prevent inadvertently recruiting the same student twice. All other anticipated verbal interactions were scripted in advance. There were no deviations from the script.

Similar recruitment procedures were previously used in this setting [26]. Those agreeing to participate were handed a sealed envelope, containing either the information sheet and informed consent form plus the AUDIT or the AUDIT alone. The time taken for study participation did not exceed 10 minutes in any case, and in most cases it was around 5 minutes or less. Upon completion of the form(s), the participants enclosed them in another envelope that was provided. The fieldworker was unaware of the participant’s assignment, i.e. the contents in the envelope. The study was entirely anonymous and confidential (names were not required) and other socio-demographic or other data were not collected.

### Sequence generation and allocation concealment

An author not involved in fieldwork (PK) generated a sequence of 100 random numbers from 1-100 using the random sequence generator available on www.random.org/sequences. Two sets of similar envelopes were then prepared; the ‘a’ set (the intervention group) contained the participant information sheet, the informed consent form and the AUDIT questionnaire, while the ‘b’ set (the comparator group) only contained the AUDIT questionnaire. 50 envelopes were prepared for each group, and returned to the research fieldworker (LF). This process was repeated for the main phase. Both LF and the participants were unaware of their assignment, until opening the envelope, thus ensuring adequate allocation concealment.

### Sample size

We undertook the trial in two phases. In the run-in phase (RP), we recruited 100 participants, 50 in each group. The study protocol required a cessation of fieldwork at this point, analyses of the data, with a view to deciding whether or not to proceed with a full scale trial. We would not have carried on if it appeared futile to do so, or if any difference at that point was too small to detect within our resources. We declared an intention to publish, regardless of whether the study proceeded further.

At the end of the run-in phase, when 98 participants (49 in each group), were analysed, there was a difference in the primary outcome between the two groups, although it did not reach statistical significance, and was in the opposite direction to that hypothesised (see Table 1). There was also a statistically significant difference on the secondary outcome at this point, again in the opposite direction to that which had been hypothesised (see Table 2). Hence, we decided to pursue a fully powered trial, with the main phase (MP) power calculation based on the mean difference in AUDIT scores (the primary outcome) between the two groups at the end of the run-in phase. Based on means of 8.1 and 6.5 with standard deviations of 5.1 and 4.4 in the intervention group (IG) and comparator group (CG) respectively, 187 participants were needed in each group (374 in total) in order to provide 90% power to detect a statistically significant difference between the two groups. Tests were two-sided, with p-values equal to or less than 0 · 05 judged as being significant. An additional 280 participants were thus recruited in the main phase.

**Table 1:** **Recruitment profile of participants by phases**

**Phases**	**Contacted**	**Screening1** ^**a**^	**Screening2** ^**b**^	**Screening3** ^**c**^	**Successfully recruited**	
					**IG**	**CG**
**Run-in**	214	184 (86)	106 (50)	109 (51)	50 (23)	50 (23)
**Main**	488	378 (78)	306 (63)	300 (62)	140 (29)	140 (29)
**TOTAL**	702	562 (80)	412 (59)	409 (58)	190 (27)	190 (27)

^a^“are you a student?”; ^b^“do you drink alcohol?”; ^c^“would you like to take part in a brief health survey, which will take around 5 minutes?”. All those who passed these three screening stages provided dates of birth and were successfully recruited.

**Table 2:** **Primary and secondary outcome by group and phases**

**Outcomes**	**AUDIT score**				**AUDIT 8+**			
**Phases**	**IG**	**CG**	**Mean difference (95% CI)**	**p-value**	**IG**	**CG**	**OR (95% CI)**	**p-value (Chi** ^**2**^ **)**
	**Mean (SD)**	**Mean (SD)**			**N (%)**	**N (%)**		
**Run-in**	8.1 (5.1)	6.5 (4.4)	-1.57 (-3.50, 0.36)	0.12	24 (49)	14 (29)	2.4 (1.04, 5.53)	0.04
**Main**	9.1 (5.9)	10.0 (6.6)	0.94 (-0.54, 2.42)	0.21	77 (55)	78 (56)	0.97 (0.61, 1.56)	0.91
**Combined**	8.8 (5.7)	9.1 (6.3)	0.29 (-0.94, 1.51)	0.65	101 (53)	92 (49)	1.21 (0.81, 1.81)	0.36

### Data analysis

The primary outcome was the mean AUDIT score. The secondary outcome was the proportion of participants in each group scoring 8 or more on the AUDIT. The analyses of these outcomes were undertaken according to a pre-established plan. The primary outcome, AUDIT score, is a continuous measure, and the mean difference was calculated and compared by a *t*-test. The secondary outcome is a binary measure and was analysed using chi-squared tests. Odds ratios (OR) were also calculated. These analyses were undertaken at the end of run-in phase and at the conclusion of the main phase. Finally, we investigated effect modification by phase, and moderation of effects by age and gender through the inclusion of interaction terms in regression models. All analyses were undertaken in STATA (version 13).

We have adhered to the CONSORT guidelines in reporting the results of our research (see Additional file 1) [28].

## Results

**Figure 1:**
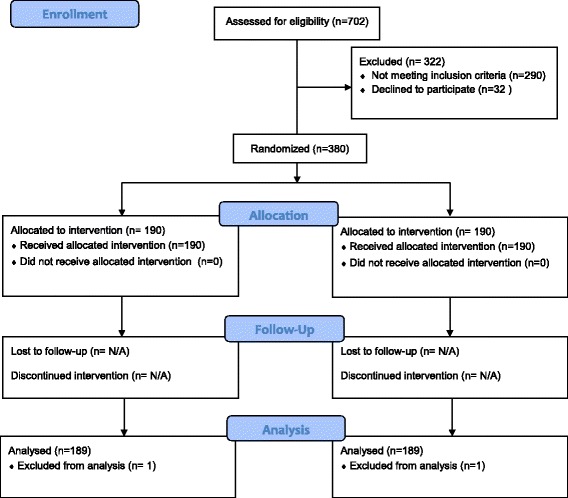
CONSORT 2010 Flow Diagram.

A total of 380 participants were successfully recruited, resulting in 190 participants in each group (Figure 1). Table 1 provides an overview of the recruitment profile of participants in the two phases. The mean age of participants was 23 years, and 58% of participants were male. In terms of age distribution, 37% were aged 21 years or younger; 52% aged between 22 and 28 years; 11% aged 29 years or older (oldest 46 years).

In the run-in phase, data from one participant in each group was incomplete and excluded. Thus, a total of 98 participants and 378 participants were analysed at the run-in phase and final trial respectively (see Figure 1). The run-in phase was conducted between 20/01/2014 and 30/01/2014 while the main phase between 24/02/2014 and 14/03/2014.

### Primary outcome

There is no evidence of any statistically significant difference in the mean AUDIT score between groups (see Table 2). There is, however, good evidence to suggest that the mean AUDIT score, for all participants combined, is significantly lower in the main phase 7.3 (SD = 4.8) compared to that in the run-in phase 9.6 (SD = 6.3) (p = 0.001). Additionally, there is weak evidence of an interaction effect of the groups by different phases on the AUDIT score (p = 0.065, see Table 3), i.e. that the mean AUDIT score obtained in the main phase was different to the mean score obtained in the run-in phase. This suggests that participants enrolled in the main- phase responded differentially compared to participants enrolled in the run-in phase. The reasons for this differential response are further explored in the discussion section. There was no evidence of interactions of groups by gender or by age categories on the primary outcome (see Table 3).

**Table 3:** **All interactions tested between groups and possible moderators of outcomes**

Outcomes*	**Phases**		**Gender**		**Age 22-28 years**		**Age 29 years or older**	
	**Summary measure**	**p-value**	**Summary measure**	**p-value**	**Summary measure**	**p-value**	**Summary measure**	**p-value**
**AUDIT score** (Coefficient, 95% CI)	-1.24, 95% CI -5.77, 3.29	0.59	-3.12, 95% CI -7.13, 0.90	0.13	-1.04, 95% CI -5.34, 3.26	0.63	-0.10, 95% CI -6.93, 6.74	0.98
**AUDIT 8 +** (OR, 95% CI)	2.47, 95% CI 0.95, 6.44	0.065	1.05, 95% CI 0.45, 2.46	0.91	0.78, 95% CI 0.33, 1.88	0.59	1.86, 95% CI 0.40, 8.64	0.43

*The reference categories are run-in phase, male and aged 21 or younger.

### Secondary outcome

In the run-in phase, participants belonging to the intervention group were more than twice as likely to have an AUDIT 8+ score compared to participants belonging to the comparator group (see Table 2). However, this was not the case in the main phase or when both the phases were combined. The interaction test demonstrated weak evidence of an interaction of groups by phase on the secondary outcome (see Table 3), indicating that the differences between the groups observed in the run-in phase compared to the main phase could not have occurred by chance. The reasons for this differential response are further explored in the discussion section. There was no evidence of an interaction effect by gender or by age categories on AUDIT 8 +.

## Discussion

The present study suggests that there is no strong evidence of an effect of the completion of a standard informed consent procedure on self-reported drinking behaviour and consequences as measured by the AUDIT in comparison with the absence of such a procedure. However, there was striking variability in the values of both the primary and secondary outcomes between the two phases of the trial, and most importantly for the present study, in the differences between the groups. The AUDIT questionnaire is considered to have good psychometric properties in terms of validity and reliability [27,29]. So it is likely that factors extrinsic to the questionnaire are relevant to the differences between the phases. These could relate to the participants such as socio-demographic characteristics, the settings such as the specific locations within institutions where the questionnaires were completed, and the educational institutions themselves, as well as timing of data collection. These factors could provide explanations of the differences in the between-group outcomes observed between the two phases of the trial.

As the study used a randomised controlled trial design, it can be assumed that individual factors, for example relating to socio-demographics and educational status are balanced across both groups [30] and that sources of differences between groups other than the experimental contrast within each phase may arise only by chance. The group by phase interaction data suggest that the differences between the groups in the run-in compared to the main phase are, however, unlikely to have occurred by chance. The lack of moderation by age or gender indicates that the main effects of the experimental contrast are similar for these variables. Explanations relating to settings are more appropriate in this context, in that different institutions were used in each phase, and there were differences in the fieldwork locations within institutions. Fieldwork differences are also reflected in the proportions of successful recruitments at the three sites, the highest being at ULU, which was the site in the main phase. In the run-in phase, participants were contacted at two sites, the students’ union bar at one site (SOAS) while either in the lounge area adjoining the café or in the refectory at Goldsmiths College. In the main phase, all the participants were contacted at one site (ULU), either in the lounge area near the café or in the students’ union bar. Although we did not record the precise location of student contact, we estimate that over two thirds of the recruitment at ULU took place in the students’ bar and nearly all of them were either having a drink or were queuing to buy one. Impact on reporting behaviour may well be influenced by this context, particularly as there is evidence available that completing alcohol questionnaires in student bars produces different responses compared with libraries [31]. It is a clear limitation of this study that we do not have data on the precise location of recruitment and study completion. The possible implication is that differences in reporting behaviour induced by the informed consent procedure do exist in non-bar settings, whilst in bars, contextual factors may eliminate differences in reporting behaviour that will be found elsewhere. It is thus an important incidental finding that alcohol studies which ask participants about their drinking and its consequences in bars may be subject to very different dynamics affecting reporting behaviour than in other settings. The lack of moderation by age or gender, arguably strengthens these observations about the possible effects of settings on reporting behaviour.

Another limitation of the study is that we are unable to determine if the participants assigned to the intervention group actually read the information sheet prior to signing the consent form. Although we could have adopted strategies such as checking the actual time a participant took to complete the questionnaire, or adding a brief question or two, as surrogate measures of having read the information sheet, such possibilities are fraught with measurement complexity and must be carefully designed to avoid interfering with the planned experimental contrast. Nonetheless, it is interesting to consider the prospect of using existing research situations in which participants are encouraged to read carefully the information sheet (possibly also where they are aware that there is some sort of check that they have done so), to extend the present study. Such scenarios make it more likely that those randomised to consent, would actually be exposed to the procedure whose effects are being evaluated. The absence of any exposure enhancement measures in the present study, also implies some degree of experimental manipulation failure, in that not all randomised participants may have been fully exposed to the possible effects we were seeking measure. This should be borne in mind when interpreting the results of the present study.

If effects of the type we hypothesised do exist, and we suggest that despite the overall finding, this study can provide some tentative evidence that they do, we may anticipate that they will vary in their magnitude, not only by setting or context, but also according to the precise circumstances of the research study and how the informed consent procedure is implemented. The fact that the differences in reporting behaviour observed in the run-in phase were in the opposite direction to that which we had hypothesised illustrates how little we know about this subject. It could be that drinking behaviour is especially complex to investigate in this regard, and studies undertaken in other areas will be informative. As well as further experiments, well conducted qualitative studies will be valuable in developing understanding of these issues.

## Conclusion

There is no strong evidence that completion of informed consent alters self-reporting behaviour on alcohol. The generalisability of this finding is contingent upon further investigations of the contexts in which such studies take place, and the effects these settings may have on reporting behaviour.

## Additional file

Additional file 1:
**CONSORT Checklist.**

## Abbreviations

AUDIT: Alcohol Use Disorder Identification Test
CI: Confidence interval
CG: Comparator group
IG: Intervention group
MP: Main phase
OR: Odds ratios
RCTs: Randomised controlled trials
RP: Run-in phase
SD: Standard deviation
SOAS: School of Oriental and African Studies
ULU: University of London Union

## References

[1] Eyal N (2014). Using informed consent to save trust. J Med Ethics.

[2] Moher D, Hopewell S, Schulz KF, Montori V, Gotzsche PC, Devereaux PJ (2012). CONSORT 2010 explanation and elaboration: updated guidelines for reporting parallel group randomised trials. Int J Surg.

[3] Weindling P (2001). The origin of informed consent: the International Scientific Commission on Medical War Crimes, and the Nuremberg code. Bull Hist Med.

[4] Smajdor A, Sydes MR, Gelling L, Wilkinson M (2009). Applying for ethical approval for research in the United Kingdom. BMJ.

[5] Torgerson DJ, Roland M (1998). What is Zelen's design?. BMJ.

[6] Zelen M (1979). A new design for randomized clinical trials. N Engl J Med.

[7] Zelen M (1990). Randomized consent designs for clinical trials: an update. Stat Med.

[8] Cordasco KM (2013). Obtaining Informed Consent From Patients: Brief Update Review. Making Health Care Safer II: An Updated Critical Analysis of the Evidence for Patient Safety Practices.

[9] Doyal L (2002). Good clinical practice and informed consent are inseparable. Heart.

[10] Brener ND, Billy JO, Grady WR (2003). Assessment of factors affecting the validity of self-reported health-risk behavior among adolescents: evidence from the scientific literature. J Adolesc Health.

[11] Durant LE, Carey MP, Schroder KE (2002). Effects of anonymity, gender, and erotophilia on the quality of data obtained from self-reports of socially sensitive behaviors. J Behav Med.

[12] Beatty JR, Chase SK, Ondersma SJ (2014). A randomized study of the effect of anonymity, quasi-anonymity, and Certificates of Confidentiality on postpartum women's disclosure of sensitive information. Drug Alcohol Depend..

[13] Noknoy S, Rangsin R, Saengcharnchai P, Tantibhaedhyangkul U, McCambridge J (2010). RCT of effectiveness of motivational enhancement therapy delivered by nurses for hazardous drinkers in primary care units in Thailand. Alcohol Alcohol.

[14] McCambridge J, Kypri K, Elbourne D (2014). Research participation effects: a skeleton in the methodological cupboard. J Clin Epidemiol.

[15] McCambridge J (2015). From question-behaviour effects in trials to the social psychology of research participation. Psychol Health.

[16] McCambridge J, Butor-Bhavsar K, Witton J, Elbourne D (2011). Can research assessments themselves cause bias in behaviour change trials? A systematic review of evidence from solomon 4-group studies. PLoS One.

[17] McCambridge J, Witton J, Elbourne DR (2014). Systematic review of the Hawthorne effect: new concepts are needed to study research participation effects. J Clin Epidemiol.

[18] McCambridge J, Kypri K (2011). Can simply answering research questions change behaviour? Systematic review and meta analyses of brief alcohol intervention trials. PLoS One.

[19] Orne MT (1962). On the social psychology of the psychological experiment: With particular reference to demand characteristics and their implications. Am Psychol.

[20] McCambridge J, de Bruin M, Witton J (2012). The effects of demand characteristics on research participant behaviours in non-laboratory settings: A systematic review. PLoS ONE.

[21] McCambridge J, Kypri K, Elbourne D (2014). In randomization we trust? There are overlooked problems in experimenting with people in behavioral intervention trials. J Clin Epidemiol.

[22] Fitzgerald JL, Mulford HA (1987). Self-report validity issues. J Stud Alcohol.

[23] Embree BG, Whitehead PC (1993). Validity and reliability of self-reported drinking behavior: dealing with the problem of response bias. J Stud Alcohol.

[24] Davis CG, Thake J, Vilhena N (2010). Social desirability biases in self-reported alcohol consumption and harms. Addict Behav.

[25] Babor TF, Higgins-Biddle JC, Saunders JB, Monteiro MG (2001). The Alcohol Use Disorders Identification Test. Guidelines for Use in Primary Care.

[26] McCambridge J, Day M (2008). Randomized controlled trial of the effects of completing the Alcohol Use Disorders Identification Test questionnaire on self-reported hazardous drinking. Addiction.

[27] Selin KH (2003). Test-retest reliability of the alcohol use disorder identification test in a general population sample. Alcohol Clin Exp Res.

[28] Schulz KF, Altman DG, Moher D, Group C (2010). CONSORT 2010 statement: updated guidelines for reporting parallel group randomised trials. BMJ.

[29] Meneses-Gaya C, Zuardi AW, Loureiro SR, Crippa JAS (2009). Alcohol Use Disorders Identification Test (AUDIT): an updated systematic review of psychometric properties. Psychol Neurosci.

[30] Altman DG, Bland JM (1999). Treatment allocation in controlled trials: why randomise?. BMJ.

[31] Cooke R, French DP (2011). The role of context and timeframe in moderating relationships within the theory of planned behaviour. Psychol Health.

